# The effect of different activation irrigations on intracanal smear layer removal: a vitro study

**DOI:** 10.3389/fbioe.2024.1507525

**Published:** 2024-12-04

**Authors:** Lingxiang Wang, Bo Feng, Shaojing Shi, Degang Sun, Di Wu

**Affiliations:** Department of Cariology and Endodontology, Qingdao Stomatological Hospital Affiliated to Qingdao University, Qingdao, Shandong, China

**Keywords:** smear layer, ultrasonic technique, ER, Cr, YSGG laser, EDDY activation technique, scanning electron microscope

## Abstract

**Objective:**

To compare the effect of syringe irrigation technique, passive ultrasonic activation technique, EDDY activation technique and Er,Cr,YSGG laser activation technique on smear layer removal in root canals *in vitro*.

**Methods:**

Forty mandibular first premolars with single canal were collected from patients in Qingdao Stomatological Hospital affiliated to Qingdao University. After root canal preparation with ProTaper Universal to F3, they were randomly divided into four groups (n = 10) according to different activation irrigations for the final washing: syringe irrigation (SI), passive ultrasonic activation (PU), EDDY activation (EDDY) and Er,Cr,YSGG laser activation (YSGG). Finally, all the crowns of them were cut off and the root length was trimmed to 15 mm. The roots were split longitudinally and observed with scanning electron microscope (SEM) for assessment of smear layer removal in different parts of the root canal.

**Results:**

All groups showed similar effects for cleaning the root canals in the coronal thirds (P > 0.05). For cleaning the root canals in the middle thirds, PU group, EDDY group and YSGG group showed similar effects, (P> 0.05). They were more effective than SI group (P < 0.05). For cleaning the root canals in the apical thirds, PU group and EDDY group showed similar effects (P> 0.05). They were more effective than SI group (P < 0.05). YSGG group was more effective than other groups (P < 0.05).

**Conclusion:**

Er,Cr,YSGG laser activation technique can remove smear layer of root canals effectively. The cleaning effect of the passive ultrasonic activation technique, EDDY activation technique is better than that of syringe irrigation technique.

## 1 Introduction

Infection control is the core of root canal treatment. At present, the infection control of root canal mainly depends on the combination of mechanical and chemical preparation of the root canal. The mechanical preparation of a root canal results in a large amount of smear layer (thickness of 2–5 μm) formation mixed with inorganic calcified tissues, organic matrix, and dentinal debris. Smear layer formation leads to a number of unfavorable consequences such as blocking the surface of dentinal tubules as well as the penetration of irrigants, medications, and filling materials into the dentinal tubules (Widbiller et al., 2021; Chavate et al., 2024). In endodontics, there is a great emphasis on removal of the smear layer and using different intra-canal irrigators. However, studies have shown that the traditional syringe irrigation technique is difficult to effectively remove the smear layer of the root canal wall, especially the root apex section (Xinyu and Xue, 2022; Drukteinis et al., 2024). Therefore, the use of the new auxiliary root canal irrigation activation technology has important clinical significance.

Kinetic energy irrigation technique can transfer different forms of energy to the root canal solution, which can effectively and fully transport the solution to the root canal system, improve the depth of its entry into the root canal, and activate the active components (Kaur et al., 2024). In recent years, the kinetic energy irrigation technique include sonic, ultrasonic, and laser-activated irrigation and so on. The traditional syringe irrigation technology is difficult to effectively deliver the solution to the root tip area due to the “air lock effect” in the apical area (Haupt et al., 2020). The sonic and ultrasonic activated irrigation technique can play a role through acoustic-streaming-effect, unltrasonic cavitation effect and thermal effect. For example, VDW’s EDDY sonic device can operate at a frequency of 6 kHz and an amplitude of 346 μm (Tomson and Simon, 2016), thus achieving the purpose of deeper removal of smear layers and tissue debris. Different kinds of laser-activated irrigation can be achieved by acoustic-streaming-effect, unltrasonic cavitation effect and thermal effect. The steam bubbles can be formed in their working process, and the volume change caused by the bubble bursting can cause the movement of the root tube flushing liquid, so as to achieve a better cleaning effect of the smear layer (Meire and De Moor, 2024).

Therefore, the aim of the present study was to evaluate the effectiveness of the syringe irrigation technique, passive ultrasonic activation technique, EDDY activation technique and Er,Cr,YSGG laser activation technique on smear layer removal in root canals during chemomechanical preparation.

## 2 Materials and methods

### 2.1 Materials

This experimental study was performed on caries-free mandibular first premolars (n = 40) extracted due to orthodontic treatment need. The extracted teeth were examined carefully to ensure that they met the following criteria (Grischke et al., 2014): fully developed teeth with a completely closed apex, length of the teeth ranging from 20 to 25 mm, and the root length greater than 10 mm. Any teeth treated endodontically or presenting with dysplasia, calcification, or root resorption were excluded. The Institutional Ethics Research Committee (NO.2023KQYX037) approved the design of this study, and an informed written consent to participate was obtained from all patients. All teeth were cleaned and stored in 0.9% normal saline at 40°C until further experimentation.

The irrigation reagents used in the present study included NaOCl (Chlorex, Durham, United Kingdom), EDTA gel and solution (17%) (Chlorex, Durham, United Kingdom). The instruments used in the present study included X-Smart motor, K-type files, ProTaper Universal (Denstply-Maillefer, Ballaigues, Switzerland), 27-gauge Monoject endodontic needles (Ultradent, South Jordan, UT, United States), sonic S motor, EDDY (VDW, Germany), K25-21 ultrasonic file (Acteon, France), Er,Cr,YSGG waterlaser (Biolase, San Clemente, America) and scanning electron microscope (Vega3 Twscan, Czech Republic).

### 2.2 Specimen preparation

Pulp tissue remnants were removed from each root canal with fine, barbed broaches (Maillefer, Ballaigues, Switzerland) before the biomechanical preparations. An ISO#15 K-type file was inserted into the root canal until it was visible at the apical foramen, and the working length of each root canal was established at 1 mm from the apical foramen. All teeth were prepared using ProTaper Universal to F3. During the canal preparation, the canals were prepared using 17% EDTA gel and irrigated with 2 mL of 2% NaClO irrigants between each filing.

All mandibular first premolars were divided randomly into four groups (n = 10 for each group). The canals were irrigated as detailed in Table 1, and irrigation with 2 mL of normal saline was performed between each irrigant.

**TABLE 1:** Description of the various study groups and the corresponding irrigation treatments.

Group	Preparation	Final washing
SI group	17% EDTA gel +2% NaOCl	17% EDTA solution + SI+ 2% NaOCl
PU group	17% EDTA gel +2% NaOCl	17% EDTA solution + PU+2% NaOCl
EDDY group	17% EDTA gel +2% NaOCl	17% EDTA solution + EDDY+2% NaOCl
YSGG group	17% EDTA gel +2% NaOCl	17% EDTA solution + YSGG+2% NaOCl

Syringe irrigation (SI): a 27-gauge Monoject endodontic needle attached to a Luer-Loc syringe with 17% EDTA solution were placed 2 mm away from the apical stop and washed the whole root canal at a constant and slow speed for 15 s. The needle was then used to wash the root canal with 2% NaClO solution for 15s.

Passive ultrasonic activation (PU): 17% EDTA solution was pre-injected into the root canal and the K25-21 was placed 2 mm away from the apical stop and washed the whole root canal at a constant and slow speed for 15 s. The K25-21 was then used to wash the root canal with 2% NaClO solution for 15s.

EDDY activation (EDDY): 17% EDTA solution was pre-injected into the root canal and the EDDY (25#06) was placed 2 mm away from the apical stop and washed the whole root canal at a constant and slow speed for 15 s.The EDDY was then used to wash the root canal with 2 %NaClO solution for 15s.

Er,Cr,YSGG laser activation (YSGG): 17% EDTA solution was pre-injected into the root canal. Set the water laser operator to the root canal treatment mode (H mode) and place the RFT2 and RFT3 laser fiber (power 0.75W, 20% air, 30% water, pulse frequency 20 Hz) 2 mm away from the apical stop and washed the whole root canal at a constant and slow speed for 15 s. The laser fibers were then used to wash the root canal with 2% NaClO solution for 15s.

Finally, the teeth were rinsed free of agents with normal saline and transferred to the fixative (2.5% glutaraldehyde).

### 2.3 Scanning electron microscopy (SEM) and debris scoring

**FIGURE 1:**
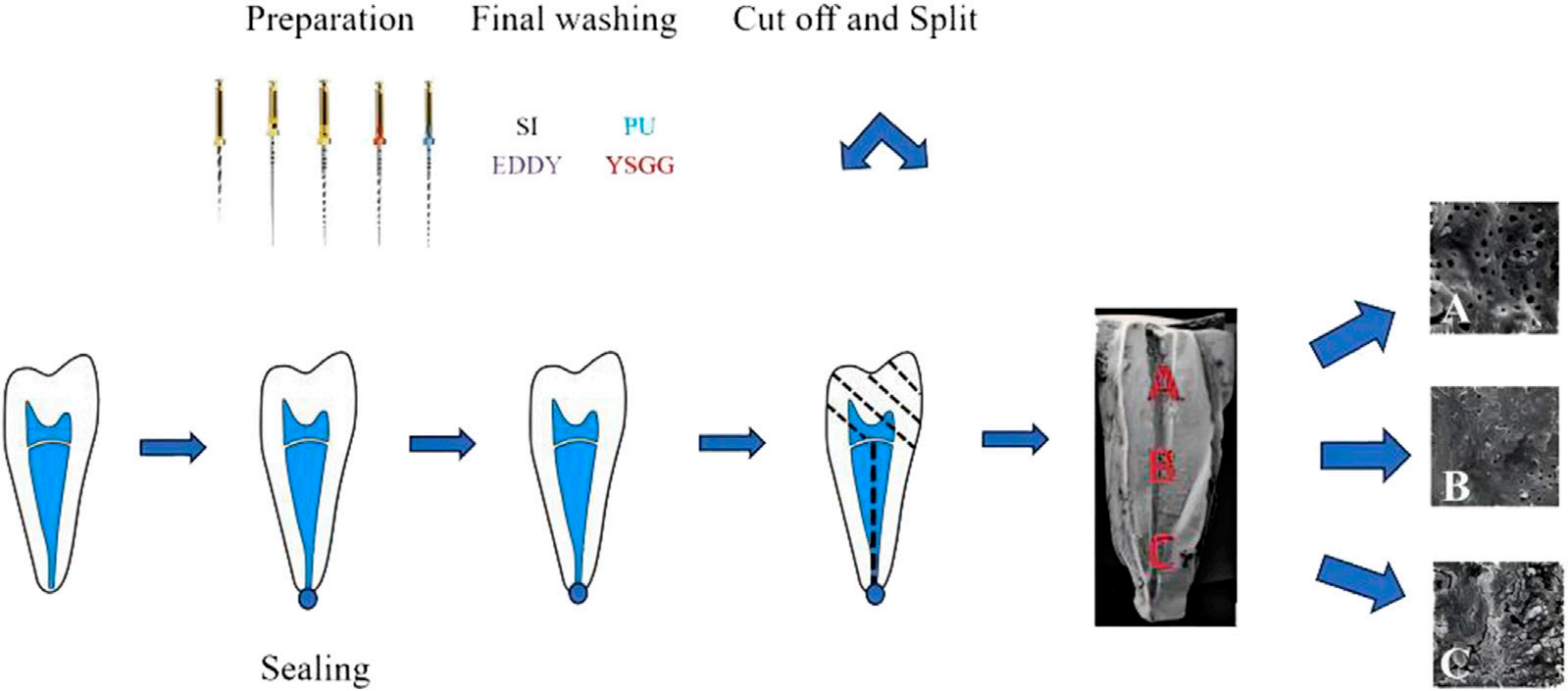
Schematic presentation of the fragment preparation process and the various segments. **(A)** Coronal third, **(B)** middle third, **(C)** apical third.

All roots were removed from the fixative, and gutta-percha cones were inserted into the root canals. The objective was to avoid any intrusion of the cutting disc into the canals, which would pollute the samples by splattering cutting debris into the root canal system.To yield root specimens of uniform length (15 mm), the teeth were decoronated at the level of the cementoenamel junction using a bone chisel and hammer. The grooves were demarcated by a diamond disk along the buccal and lingual surfaces to mark three parts: coronal, middle, and apical thirds. The roots were then split into two halves with a hammer and a microtome blade. For each root, the half containing the most visible prepared parts were used in the study, returned to fresh fixative solution (2.5% glutaraldehyde), and incubated overnight at 4°C. The specimens were rinsed with sterile water and sequentially dehydrated using a gradient of ethanol (30%, 50%, 70%, 80%, 90%, and 100%, v/v) at 15-min intervals. The dehydrated specimens were transferred to a critical point dryer (Tsousimis Autosamdri®-815 Series A, United States) with absolute alcohol as the intermediate fluid and liquid CO_2_ as the transition fluid. Following mounting and gold sputter coating (EikoIB-3 ion sputter coater, Japan), the surface morphology of the specimens was analyzed using SEM (Vega3 Twscan, Czech Republic). At the observation stage, the canal walls in the apical, middle, and coronal thirds were examined, and photomicrographs of representative areas were taken at ×2000 magnification as in Figure 1.

The quantitative scoring of the canal wall debris was evaluated using the protocol described by Faras et al. (2016), as follows: 1. No smear layer and open dentinal tubules; 2. A small amount of smear layer and open dentinal tubules; 3. A thin smear layer and partially open dentinal tubules; 4. Partial covering of dentinal tubules with a thick smear layer; 5. Full covering of dentinal tubules with a thick smear layer.

### 2.4 Statistical analysis

All statistical analyses were performed with SPSS 26.0 software (IBM-SPSS Inc., Chicago). First, the full set of samples was independently and blindly evaluated by two observers, and Cohen K scores were calculated to determine the inter-examiner reliability. Second, the debris scores for different irrigants were analyzed by the nonparametric Kruskal–Wallis test and the Mann–Whitney rank sum test for pairwise comparisons. The level of statistical significance was set at P < 0.05 (Do and Gaudin, 2020).

## 3 Results

**FIGURE 2:**
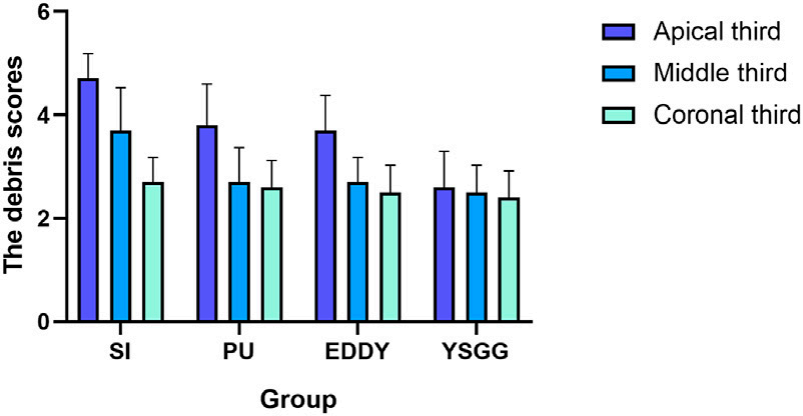
Scores for smear layer removal in all groups according to the quantitative scoring of the canal wall debris by Faras et al. (2016). SI = syringe irrigation, EDDY = EDDY activation, PU = passive ultrasonic activation, YSGG = Er,Cr,YSGG laser activation.

In terms of the root canal sections, a similar trend was observed for four groups in the coronal thirds (P > 0.05). In the middle thirds: the PU group, the EDDY group and the YSGG group had a similar effective (P > 0.05), but they cleaned significantly (P < 0.05) better than the SI group. In the apical thirds: the PU group and the EDDY group had a similar effective (P > 0.05), but they cleaned significantly (P < 0.05) better than the SI group. The YSGG group was significantly (P < 0.05) more effective than all of the other groups (Figure 2).

**FIGURE 3:**
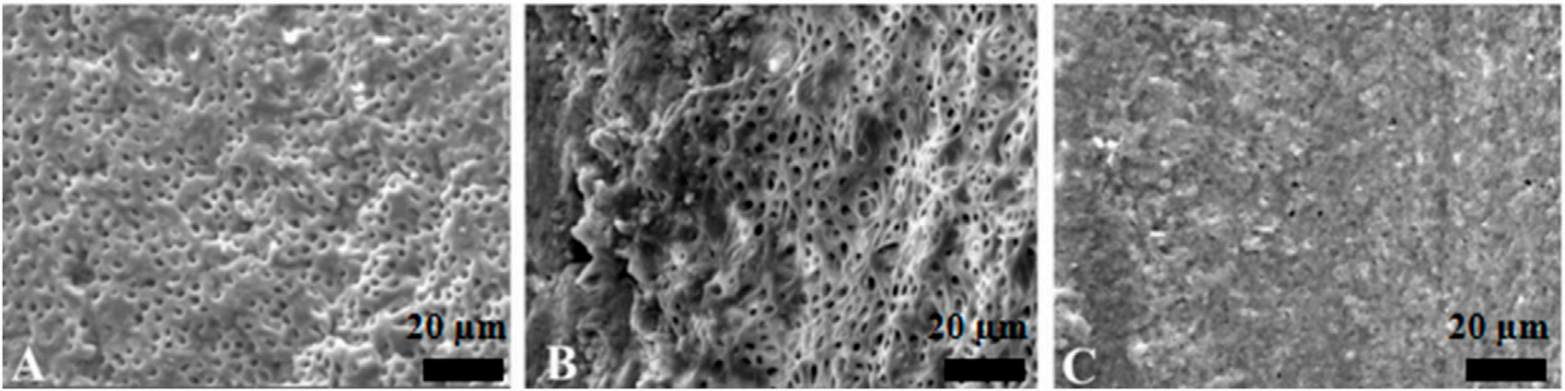
Representative scanning electron microscopy images showing the surface morphology in syringe irrigation group. The opening of dentinal tubules as a result of debris removal is apparent in the coronal **(A)** and middle **(B)** thirds, and it was less effective in the apical thirds **(C)** of the root canals (scale bar = 20 µm).

**FIGURE 4:**
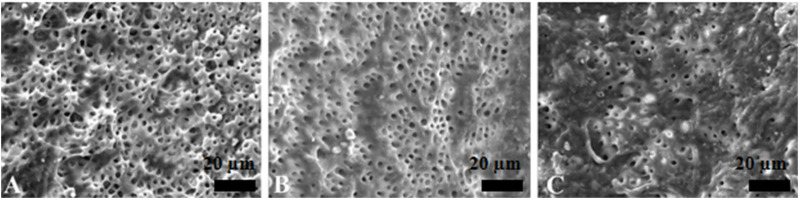
Representative scanning electron microscopy images showing the surface morphology in passive ultrasonic irrigation group. The opening of dentinal tubules as a result of debris removal is apparent in the coronal **(A)** and middle **(B)** thirds, and it was less effective in the apical thirds **(C)** of the root canals (scale bar = 20 µm).

**FIGURE 5:**
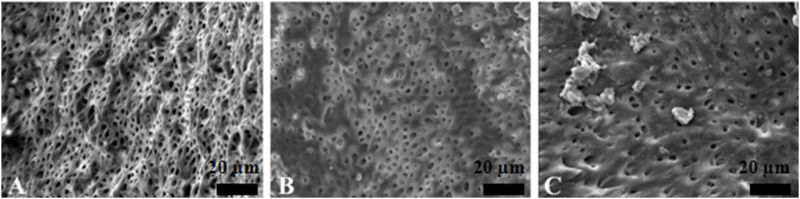
Representative scanning electron microscopy images showing the surface morphology in EDDY irrigation group. The opening of dentinal tubules as a result of debris removal is apparent in the coronal **(A)** and middle **(B)** thirds, and it was less effective in the apical thirds **(C)** of the root canals (scale bar = 20 µm).

The PU group and the EDDY group had a similar effect of activation irrigations on intracanal smear layer removal, better than the SI group (Figures 3–5).

**FIGURE 6:**
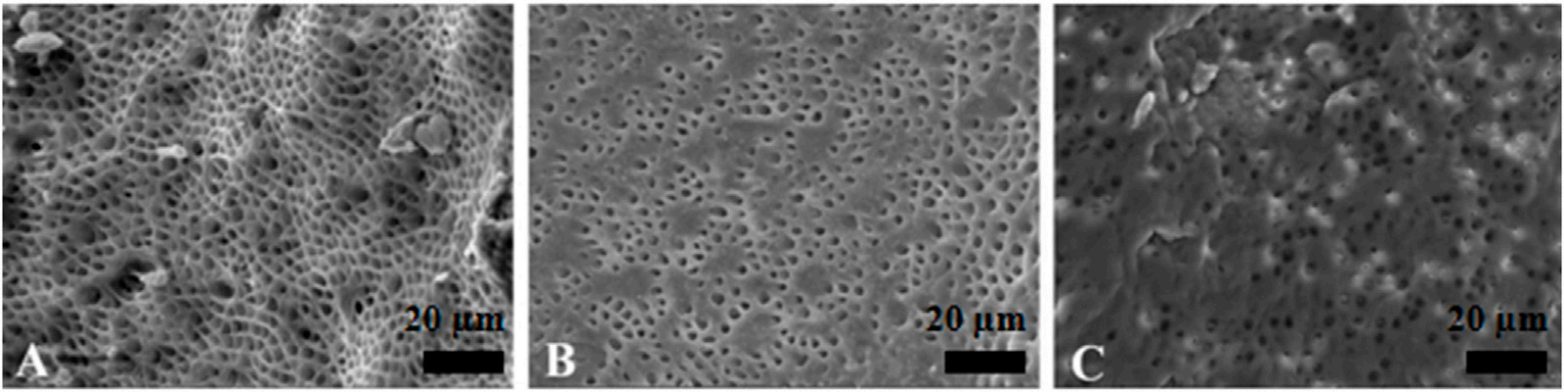
Representative scanning electron microscopy images showing the surface morphology in Er,Cr,YSGG laser irrigation group. The opening of dentinal tubules as a result of debris removal is apparent in the coronal **(A)** and middle **(B)** thirds, and it was less effective in the apical thirds **(C)** of the root canals (scale bar = 20 µm).

The YSGG group was significantly more effective than all of the other groups, showing that Er,Cr,YSGG laser irrigation has a better capability to remove the canal debris and to open the dentinal tubules (Figure 6).

## 4 Discussion

The main purpose of root canal treatment is to remove the infection, which is mainly caused by microbial infection and the smear layer generated during root canal preparation (Iandolo et al., 2020). The 2∼5 μm thick smear layer will cover the root canal wall and block the dentin tubules, and the filling material will not be in close contact with the root canal wall, which will affect the root canal treatment effect (Chavate et al., 2024). In order to effectively remove root canal infection, root canal preparation devices and chemical preparation drugs have been updated, but studies have shown that preparation devices can only reach about 50% of the root canal anatomy, and those small, narrow structures remain inaccessible (Li et al., 2020). At present, there is no ideal irrigation agent, because sodium hypochlorite can sterilize and dissolve organic matter and necrotic tissue in the root canal, and EDTA can soften and dissolve inorganic matter in the smear layer, so they are often used in combination in clinical practice (Dentistry, 2019). However, traditional irrigators have the limitation of not being able to fully deliver the irrigation to the anatomically complex areas. In addition, due to the tapered shape of the root canal system, there is an “airlock effect” within the root canal system, which prevents the irrigation agent from entering the apical thirds (Rodrigues et al., 2021). Therefore, improving the distribution of the irrigation agent so that it is adequately irrigated throughout the root canal system is essential for the effectiveness of the irrigation.

In recent years, kinetic irrigation has received extensive attention, which can transfer energy into the irrigation agent, deliver the irrigation agent more comprehensively into the root canal system, improve the distribution of the irrigation agent, and activate the active ingredients of the irrigation agent to make it work (Betancourt et al., 2020). Kinetic energy flushing makes up for the shortcomings of traditional flushing devices and plays a role in both chemical and mechanical aspects. The chemical effect of root canal irrigation is mainly achieved by sodium hypochlorite, while the mechanical effect is mainly achieved by the shear stress exerted by the flowing irrigation agent on the biofilm and residual tissue debris (Boutsioukis et al., 2022). Sonic and ultrasonic kinetic energy flushing devices work through acoustic flow effects, cavitation effects, and thermal effects, and their flushing effects have been demonstrated in several studies (Dashtimoghadam et al., 2020; Klyn et al., 2010). The intensity of the sound flow depends mainly on the speed of the sound flow, which is closely related to the frequency and amplitude of the working tip (Ahmad et al., 1988). The ultrasound working tip typically operates at a frequency of 30 kHz and an amplitude of 75 μm (Boutsioukis and Arias, 2022), while the EDDY operating tip operates at a frequency of 6 kHz and an amplitude of 346 μm. The motion of the ultrasonic working tip is a plane, and the motion of the EDDY rotates around its axis in a three-dimensional space (Tomson and Simon, 2016). In my experiment, there was no significant difference in the score of the smear layer between the four groups in the coronal thirds, which proved that the four groups of auxiliary irrigation technology could achieve good cleaning effect in the thick region. In the middle thirds and the apical thirds, the cleaning effect of the passive ultrasonic group, the EDDY group, and the YSGG group were better than that of the syringe irrigation group, because of their agitation activates the system to accelerate the flow of fluid in the root canal, which can effectively break through the area of “airlock effect”, and the resulting temperature rise can activate the activity of sodium hypochlorite, which is consistent with the results of other studies (Wimonchit et al., 2002). Therefore, kinetic energy flushing equipment is more effective than traditional irrigators when cleaning irregular, deep areas. The results of a meta-analysis study (Virdee et al., 2018) also support the idea that kinetic irrigation can create cleaner root canals compared to traditional irrigation. There was no significant difference in the cleaning effect of the smear layer in the comparison between passive ultrasonic group and EDDY group, which may be due to the large amplitude of EDDY washing, which exceeded the diameter of the root canal, and frequent tube wall contact would reduce its efficiency (Urban et al., 2017).

Lasers are characterized by low pain, minimally invasiveness, and less bleeding, and have become an important treatment tool in clinical practice in recent years. The wavelength of the Er,Cr,YSGG laser is 2.78 μm, which is close to the absorption peak of water (3 μm) (Kirmali et al., 2015). It can be found from the results of this experiment that Er,Cr,YSGG laser showed the best cleaning effect of the smear layer in the whole section of the root canal. Because when the laser energy is transferred to the coaxial air-water mixture, water mist particles can be generated in the atomization area at the front of the treatment handle, because these water mist particles carry the energy imparted by the laser, and can release energy from the reaction zone 1–2 mm away from the tip of the laser to make them have high-speed kinetic energy and rapidly expand in volume, the high-energy water molecules destroy the target cells and break through the “airlock effect” area of the root apical segment, so that the irrigation solution can fully flow in the root canal system to achieve the effect of disinfection and removal of the smear layer (Blanken et al., 2009). It has been reported (Dentistry, 2019; Betancourt et al., 2019) that the application of Er,Cr,YSGG laser in the root canal can melt the inorganic components in the dentin, which can effectively remove debris and dirt from the root canal wall. Rosalin and Yosvimol Kuphasuk (2017) took transparent isolated teeth as the research object, and used Er,Cr,YSGG laser (1.5 W, 20 HZ, 30% water, 50% air) to wash the root canal and then perform root canal filling to fill more lateral root canals and root canal isthmus, indicating that the Er,Cr,YSGG laser can effectively remove the smear layer and open the lateral branch root canal. In addition, studies have shown that the Er,Cr,YSGG laser treatment system has less smear layer and no charring phenomenon after irradiation of the tooth surface, and has a low chance of thermal damage to periodontal tissues (Asnaashari et al., 2022). Although the laser has obvious advantages, there are also some shortcomings that need our attention. Widbiller M et al. found that the surface disintegration of root canal dentine was observed with the additional activation of EDTA and particularly after laser-based techniques (Widbiller M et al., 2023). Even though it was not observed in our study, it cannot be excluded that thermal effects may still occur locally, which should be considered as a drawback for this method of activation.

This experiment is based on several commonly used methods for removing the smear layer after root canal preparation in clinical practice. The aim is to preliminarily evaluate the advantages and disadvantages of different treatment techniques, provide initial guidance for future clinical work, and offer directions for further research in the future. Currently, various methods for studying the smear layer and observing dentine tubules are emerging, such as SEM, AFM, and COSM (De-Deus et al., 2011). These methods excel in certain aspects (e.g., imaging speed, quantitative analysis, data statistics, longitudinal comparison), each with its own advantages and disadvantages. However, there is still no ideal experimental model. Although SEM has certain limitations, such as qualitative comparison, slow imaging speed, operator bias, and field of view restrictions, it remains the most commonly used method for obtaining information on the surface of dentin at this stage, as evidenced by multiple relevant literature reviews. Considering the experimental objectives and methods of this study, quantitative analysis was not involved. Instead, the focus was on comparing the effectiveness of different techniques in removing the smear layer. To minimize errors, multiple sites were evaluated using a double-blind method with two trained evaluators. Some studies have shown that SEM is difficult to distinguish between the smear layer and sclerotic dentin, which is a physiological change that occurs with age (Dewi et al., 2020; Sudhakar et al., 2023). Therefore, young teeth extracted for orthodontic purposes were selected in this study to reduce the interference of sclerotic dentin.

## 5 Conclusion

In summary, when facing the complex area of the root canal, it is necessary not only to have an effective irrigation solution but also to combine kinetic irrigation to achieve a more ideal irrigation effect. The root canal system is complex and changeable, so kinetic irrigation is indispensable for root canal treatment.In clinical application, the appropriate kinetic energy irrigation method should be selected according to the actual situation of the affected tooth, root canal infection, root canal curvature, and patient’s opening degree, so as to provide guarantee for the efficacy of root canal treatment.

## Data Availability

The raw data supporting the conclusions of this article will be made available by the authors, without undue reservation.

## References

[B1] Ahmad M. Ford T. R. P. Crum L. A. Walton A. J. (1988). Ultrasonic debridement of root canals: acoustic cavitation and its relevance. J. Endod. 14, 486–493. 10.1016/s0099-2399(88)80105-5 3255774

[B2] Asnaashari M. Sadeghian A. Hazrati P. (2022). The effect of high-power lasers on root canal disinfection: a systematic review. J. Lasers Med. Sci. 13, 66. 10.34172/jlms.2022.66 PMC10082900 37041778

[B3] Betancourt P. Merlos A. Sierra J. M. Arnabat-Dominguez J. Viñas M. (2020). Er,Cr:YSGG laser-activated irrigation and passive ultrasonic irrigation: comparison of two strategies for root canal disinfection. Laser Surg. 38, 91–97. 10.1089/photob.2019.4645 31397611

[B4] Betancourt P. Merlos A. Sierra J. M. Camps-Font O. Arnabat-Dominguez J. Viñas M. (2019). Effectiveness of low concentration of sodium hypochlorite activated by Er,Cr:YSGG laser against *Enterococcus faecalis* biofilm. Med. Sci. 34, 247–254. 10.1007/s10103-018-2578-6 29980946

[B5] Blanken J. De Moor R. J. G. Meire M. Verdaasdonk R. (2009). Laser induced explosive vapor and cavitation resulting in effective irrigation of the root canal. Part 1: a visualization study. Lasers Surg. Med. 41, 514–519. 10.1002/lsm.20798 19639622

[B6] Boutsioukis C. Arias-M. T. (2022). Present status and future directions–irrigants and irrigation. Int. Endod. J. 55, 588–612. 10.1111/iej.13739 35338652 PMC9321999

[B7] Boutsioukis C. Arias-Moliz M. T. Chávez de Paz L. E. (2022). A critical analysis of research methods and experimental models to study irrigants and irrigation systems. Int. Endod. J. 55, 295–329. 10.1111/iej.13710 35171506 PMC9314845

[B8] Chavate P. R. Ponnappa K. C. Nanjappa A. S. (2024). Comparative evaluation of the effect of ultrasonic and rotary agitation of herbal irrigating solutions on smear layer: a SEM study. J. Conserv. Dent. Endod. 27, 164–169. 10.4103/jcde.jcde_277_23 38463471 PMC10923225

[B9] Dashtimoghadam E. Johnson A. Fahimipour F. Malakoutian M. Vargas J. Gonzalez J. (2020). Vibrational and sonochemical characterization of ultrasonic endodontic activating devices for translation to clinical efficacy. Mater. Sci. Eng. C 109, 110646. 10.1016/j.msec.2020.110646 32228956

[B10] De-Deus G. Reis C. Paciornik S. (2011). Critical appraisal of published smear layer-removal studies: methodological issues. Oral Med. Oral Pathol. Oral Radiol. Endodontology 112, 531–543. 10.1016/j.tripleo.2011.01.046 21696983

[B11] Dentistry P. (2019). Pedodontics and preventive Dentistry, 37, 1–6.

[B12] Dewi A. Upara C. Chaiariyakul D. Louwakul P. (2020). Smear layer removal from root canal dentin and antimicrobial effect of citric acid-modified chlorhexidine. Eur. Endod. J. 5, 257–263. 10.14744/eej.2020.38258 33353912 PMC7881384

[B13] Do Q. L. Gaudin A. (2020). The efficiency of the ER: YAG laser and photon-induced photoacoustic streaming (PIPS) as an activation method in endodontic irrigation: a literature review. J. Lasers Med. Sci. 11, 316–334. 10.34172/jlms.2020.53 32802294 PMC7369550

[B14] Drukteinis S. Rajasekharan S. Widbiller M. (2024). Advanced materials for clinical endodontic applications: current status and future directions. J. Funct. Biomater. 15, 31. 10.3390/jfb15020031 38391884 PMC10889336

[B15] Faras F. Abo-Alhassan F. Sadeq A. Burezq H. (2016). Complication of improper management of sodium hypochlorite accident during root canal treatment. J. Int. Soc. Prev. Community Dent. 6, 493–496. 10.4103/2231-0762.192939 27891318 PMC5109866

[B16] Grischke J. Müller-Heine A. Hülsmann M. (2014). The effect of four different irrigation systems in the removal of a root canal sealer. Clin. Oral Investig. 18, 1845–1851. 10.1007/s00784-013-1161-6 24317958

[B17] Haupt F. Meinel M. Gunawardana A. Hülsmann M. (2020). Effectiveness of different activated irrigation techniques on debris and smear layer removal from curved root canals: a SEM evaluation. Aust. Endod. J. 46, 40–46. 10.1111/aej.12342 30907051

[B18] Iandolo A. Abdellatif D. Amato M. Pantaleo G. Blasi A. Franco V. (2020). Dentinal tubule penetration and root canal cleanliness following ultrasonic activation of intracanal-heated sodium hypochlorite. Aust. Endod. J. 46, 204–209. 10.1111/aej.12393 31846169

[B19] Kaur M. Singla M. Kaur H. Mittal L. Gupta S. Joseph M. M. (2024). Comparative evaluation of smear layer removal by using different irrigant activation techniques: an *in vitro* scanning electron microscopic study. J. Conserv. Dent. Endod. 27, 257–261. 10.4103/jcde.jcde_254_23 38634018 PMC11019803

[B20] Kirmali O. Kustarci A. Kapdan A. Er K. (2015). Effects of dentin surface treatments including Er,Cr:YSGG laser irradiation with different intensities on the push-out bond strength of the glass fiber posts to root dentin. Acta Odontol. Scand. 73, 380–386. 10.3109/00016357.2014.968872 25330165

[B21] Klyn S. L. Kirkpatrick T. C. Rutledge R. E. (2010). *In vitro* comparisons of debris removal of the EndoActivator TM system, the F file TM, ultrasonic irrigation, and NaOCl irrigation alone after hand-rotary instrumentation in human mandibular molars. J. Endod. 36, 1367–1371. 10.1016/j.joen.2010.03.022 20647098

[B22] Li Q. Zhang Q. Zou X. Yue L. (2020). Evaluation of four final irrigation protocols for cleaning root canal walls. Int. J. Oral Sci. 12, 29. 10.1038/s41368-020-00091-4 33077718 PMC7573610

[B23] Meire M. De Moor R. J. G. (2024). Principle and antimicrobial efficacy of laser-activated irrigation: a narrative review. Int. Endod. J. 57, 841–860. 10.1111/iej.14042 38340037

[B24] Rodrigues C. T. EzEldeen M. Jacobs R. Lambrechts P. Alcalde M. P. Hungaro Duarte M. A. (2021). Cleaning efficacy and uncontrolled removal of dentin of two methods of irrigant activation in curved canals connected by an isthmus. Aust. Endod. J. 47, 631–638. 10.1111/aej.12534 34097337

[B25] Rosalin H. Yosvimol Kuphasuk K. R. (2017). Effectiveness of platelet-rich fibrin in the management of pain and delayed wound healing. Eur. J. Dent. 11, 192–195. 10.4103/ejd.ejd 29279679 PMC5727738

[B26] Sudhakar S. Gupta N. Ghambir N. Singh R. Singh D. (2023). Comparative evaluation of intracanal smear layer removal by different root canal irrigants: a scanning electron microscope study. Int. J. Clin. Pediatr. Dent. 16, 633–638. 10.5005/jp-journals-10005-2648 37731794 PMC10507302

[B27] Tomson P. L. Simon S. R. (2016). Contemporary cleaning and shaping of the root canal system. Prim. Dent. J. 5, 46–53. 10.1308/205016816819304196 28826433

[B28] Urban K. Donnermeyer D. Schäfer E. Bürklein S. (2017). Canal cleanliness using different irrigation activation systems: a SEM evaluation. Clin. Oral Investig. 21, 2681–2687. 10.1007/s00784-017-2070-x 28185091

[B29] Virdee S. S. Seymour D. W. Farnell D. Bhamra G. Bhakta S. (2018). Efficacy of irrigant activation techniques in removing intracanal smear layer and debris from mature permanent teeth: a systematic review and meta-analysis. Int. Endod. J. 51, 605–621. 10.1111/iej.12877 29178166

[B30] Widbiller M. Keim L. Schlichting R. Striegl B. Hiller K. A. Jungbauer R. (2021). Debris removal by activation of endodontic irrigants in complex root canal systems: a standardized in‐vitro‐study. Appl. Sci. 11, 7331. 10.3390/app11167331

[B31] Widbiller M. Rosendahl A. Schlichting R. Schuller C. Lingl B. Hiller K. A. (2023). Impact of endodontic irrigant activation on smear layer removal and surface disintegration of root canal dentine *in vitro* . Healthc 11, 376. 10.3390/healthcare11030376 PMC9914448 36766951

[B32] Wimonchit S. Timpawat S. Vongsavan N. (2002). Scientific articles A comparison of techniques for assessment of, 1–4. 10.1097/00004770-200201000-00001 11806641

[B33] Xinyu C. Xue M. (2022). Evaluation of root-canal isthmus debridement efficacy of 3 kinds of activated irrigation technique. Hua Xi Kou Qiang Yi Xue Za Zhi 40, 554–559. 10.7518/hxkq.2022.05.008 38596976 PMC9588857

